# MultiSETTER: web server for multiple RNA structure comparison

**DOI:** 10.1186/s12859-015-0696-8

**Published:** 2015-08-12

**Authors:** Petr Čech, David Hoksza, Daniel Svozil

**Affiliations:** 1 Laboratory of Informatics and Chemistry, Faculty of Chemical Technology, University of Chemistry and Technology Prague, Technická 5, CZ-166 28 Prague, Czech Republic; 2 Department of Software Engineering, Faculty of Mathematics and Physics, Charles University in Prague, Malostranské nám. 25, CZ-118 00 Prague, Czech Republic

**Keywords:** RNA tertiary structure, Multiple RNA structure alignment, Multiple RNA structure superposition, RNA structure similarity

## Abstract

**Background:**

Understanding the architecture and function of RNA molecules requires methods for comparing and analyzing their tertiary and quaternary structures. While structural superposition of short RNAs is achievable in a reasonable time, large structures represent much bigger challenge. Therefore, we have developed a fast and accurate algorithm for RNA pairwise structure superposition called SETTER and implemented it in the SETTER web server. However, though biological relationships can be inferred by a pairwise structure alignment, key features preserved by evolution can be identified only from a multiple structure alignment. Thus, we extended the SETTER algorithm to the alignment of multiple RNA structures and developed the MultiSETTER algorithm.

**Results:**

In this paper, we present the updated version of the SETTER web server that implements a user friendly interface to the MultiSETTER algorithm. The server accepts RNA structures either as the list of PDB IDs or as user-defined PDB files. After the superposition is computed, structures are visualized in 3D and several reports and statistics are generated.

**Conclusion:**

To the best of our knowledge, the MultiSETTER web server is the first publicly available tool for a multiple RNA structure alignment. The MultiSETTER server offers the visual inspection of an alignment in 3D space which may reveal structural and functional relationships not captured by other multiple alignment methods based either on a sequence or on secondary structure motifs.

## Background

RNA structure hierarchy consists of primary, secondary and tertiary levels [1]. Primary structure is given as the sequence of four nucleotides A, U, G and C that are, within one chain, connected through a phospohodiester bond. Secondary structure is defined by base-pairing patterns and consists of canonically base-paired helices (called stems) combined with various types of non-paired regions (called loops). Secondary structure elements can be predicted from the sequence by a variety of computational approaches [2–5]. The overall 3D structure of an RNA molecule is known as a tertiary structure and is formed by long-range interactions between distant loops stabilized by canonical and noncanonical base-pairs, base stacking, cations or weak interactions [6, 7].

The role of RNA is largely determined by its tertiary structure and the comparison of 3D RNA structures is, thus, an effective tool for studying RNA function and evolutionary relationships. Several methods and servers for pairwise RNA structure comparison and alignment exist, including ARTS [8, 9], DIAL [10], iPARTS [11], SARA [12, 13], SARSA [14], Rclick [15, 16], R3Dalign [17, 18], RASS [19], FRASS [20], SETTER [21, 22] or R3D-BLAST [23]. The extension of a pairwise alignment to the simultaneous alignment of several structures is called a multiple structure alignment. While in a pairwise structure alignment biological relationships are inferred from structure similarity, with a multiple alignment we already know that structures are biologically related and we are looking for key features preserved by evolution that are difficult to identify by a pairwise alignment.

Because of the fast evolutionary divergence of RNA molecules [24, 25] that makes it difficult to produce structurally informative sequence alignments, multiple RNA structure alignment methods represent an important tool for functional annotation and evolutionary reconstruction of non-coding RNA. However, though structural information was employed in the SARA-Coffee package to derive more informative multiple sequence alignment models [26], we are not aware of any algorithm for a direct multiple RNA structure alignment. Thus, we have developed a multiple RNA structure alignment algorithm MultiSETTER [27]. MultiSETTER uses, similarly to the multiple sequence alignment algorithm Clustal [28], a guide tree which is constructed from all-against-all pairwise comparisons produced by SETTER [21]. The guide tree is then used to progressively align RNA molecules beginning with the most closely related RNAs. The advantages of the multiple structure alignment over the multiple sequence alignment are several. Multiple structure alignment can detect motifs not conserved at the sequence level. In addition, multiple structure alignments can be easily inspected visually and to detect common motifs they, thus, do not require so much molecules as multiple sequence alignments.

In this paper, we describe the MultiSETTER web server, an updated version of the SETTER web server [22]. The MultiSETTER web server makes a MultiSETTER method for a multiple RNA structure alignment available to the wide audience of biologists and bioinformaticians. The MultiSETTER web server is free and open to all users and has no login requirements. The server allows both pairwise and multiple comparisons between structures obtained either from the PDB database [29] or supplied by the user. Both 3D graphics and summary statistics are provided to make the interpretation of the alignments easier.

## Materials and methods

### SETTER algorithm

**Fig. 1:**
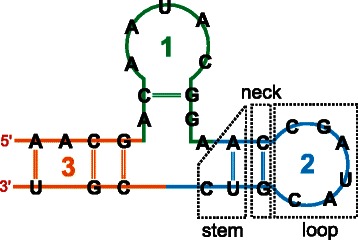
An RNA structure decomposed into three generalized secondary structure units (GSSU). Each GSSU typically consists of three parts: a stem, neck and loop. However, in some cases the loop (and, thus, also the neck) does not have to be present (GSSU 3)

In the SETTER (SEcondary sTructure-based TERtiary superposition) algorithm, RNA structures are divided into secondary-structure based fragments called generalized secondary structure units (GSSU) [21]. A generalized secondary structure unit typically consists of a stem, neck and loop (Fig. 1) and resembles, thus, a hairpin motif. After the decomposition of two structures A and B into GSSUs, each pair of GSSUs from A and B is superposed [21]. For GSSU structural superposition, SETTER uses the RMSD minimization algorithm to which 3 pairs of points (in SETTER, each residue is represented by a backbone phosphate) must be supplied. First two pairs are produced by the alignment of two corresponding neck residues. Third pair is identified by aligning each possible pair of loop nucleotides and taking the one with the lowest score. In the next step, the GSSU superposition with the lowest score is used to align whole structures. Finally, the alignment of two structures is further improved by an additional RMSD superposition of the mutual nearest neighbor residues. The aligned structures are scored by a similarity measure $\overline{S}$-distance. The statistical significance of the $\overline{S}$-distance is estimated by computing its *p*-value. The smaller the *p*-value, the more statistically significant the $\overline{S}$-distance, i.e., the more likely the alignment does not arise by a chance. The GSSU-wise superposition of RNA structures leads to low computational times while maintaining alignment accuracy comparable to several state-of-the-art RNA structure superposition methods [21].

### MultiSETTER algorithm

To align multiple RNA structures, MultiSETTER uses [27] a heuristic approach called a progressive alignment that was developed for a multiple sequence alignment [30] and that is implemented in the popular Clustal family of computer programs [28]. In MultiSETTER, each pair of RNA structures is aligned by SETTER and resulting $\overline{S}$-distances are used to construct an all-to-all distance matrix. From the distance matrix, a guide tree is calculated using the neighbor-joining method [31]. Based on a guide tree, first two most related structures are aligned and their ‘*average structure*’ is constructed by averaging positions of individual atoms. As the alignment progressively continues following a guide tree, more and more structures are averaged till the root of a guide tree is reached. Though low computational requirements of SETTER result also in a reasonable runtime of MultiSETTER, these may unacceptably grow with the size and number of aligned RNA structures. Therefore, we parallelized the following parts of SETTER and MultiSETTER:

- *Pairwise RNA structure superposition*. A pairwise superposition consists of multiple pairwise GSSU computations which can be run independently and each is, thus, assigned to a single computation unit. Only structures consisting of multiple GSSUs can take advantage of this type of parallelization.
- *Distance matrix computation.* The construction of the all-to-all distance matrix for *n* input structures requires *n* × (*n* − 1)/2 computations that can be run independently.
- *RNA merging.* The formation of an average structure from two input structures can be decomposed into the merging of aligned GSSUs [27]. The merging of GSSU pairs can be carried out independently and, thus, in parallel.

To demonstrate the influence of the parallel processing on the runtimes both of the pairwise and multiple structure alignments, we performed the pairwise alignment of two 23S large ribosomal subunits 1NKW [32] and 1S72 [33] and the multiple alignment of four 23S large ribosomal subunits 1NKW [32], 1S72 [33], 2AWB [34] and 2Y11 [35]. Each of 23S rRNA contains approximately 3000 residues. The computations were performed on the machine with 4 Cores with Hyper-V support – Intel (R) Core (TM) i7-4790 CP @ 3.60GHz, 16GB RAM, running Windows 8.1. We limited the number of threads in the Intel (R) Threading Building Blocks library between one and four. The results for both pairwise and multiple structure alignment (Table 1) demonstrate that the parallelization leads to about 2–2.5 speedup of the calculations. However, the parallelization does not scale linearly; the increase of the number of threads from three to four does not result in a significant performance gain showing the limits of the parallel processing.

**Table 1:** The influence of algorithm parallelization on SETTER and MultiSETTER runtimes

Number of threads	Pairwise alignment	Multiple alignment
1	9.3	171.4 / 466.7
2	5.7	89.9 / 257.9
3	4.7	65.0 / 194.0
4	4.4	63.4 / 190.3

Runtimes (in seconds) of the pairwise structure alignment by SETTER and multiple structure alignment by MultiSETTER as the function of the number of threads limited in the Intel (R) Threading Building Blocks library. For the multiple alignment, first number is the time needed for distance matrix computation, second number is total alignment time consisting of distance matrix computation, the creation of the average structure and the alignment of every structure against the average structure

## Implementation

The MultiSETTER web server consists of two separate applications: a core engine written in C++ that implements both SETTER [21] and MultiSETTER [27] algorithms and a web application (http://setter.projekty.ms.mff.cuni.cz) that interactively visualizes the results of a multiple structure alignment. The web application is developed in the Python Model-Template-View (MTV) web framework Django version 1.4 [36]. Server-side scripts are written in Python programming language version 2.7 [37]. For a client-side user interactivity and data visualization, jQuery JavaScript library version 1.10.4 [38] and JavaScript molecular viewer JSmol [39] are used. Data about RNA structures are stored in the SETTER database, a relational MySQL database [40] that is synchronized with the Protein Data Bank [41] every Wednesday. The SETTER database contains information such as PDB ID, chain IDs or hydrogen bonding patterns calculated by the 3DNA software [42] with its implicit settings.

## Results and discussion

### Web server

**Fig. 2:**
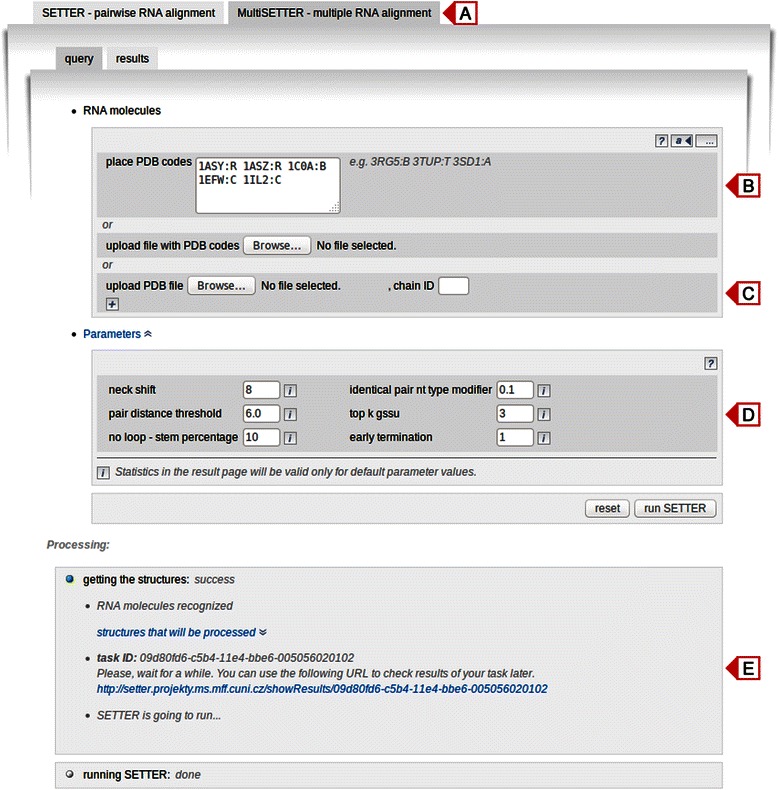
The interface of the MultiSETTER web server. **a** Selection of alignment type. **b** Input structures by their PDB IDs. **c** Input structures as user defined PDB files. **d** SETTER algorithm parameters. **e** Notification of the alignment process

Web server users can choose between two types of RNA structure alignment (Fig. 2a): pairwise (SETTER) and multiple (MultiSETTER). The SETTER part of the web server was described in detail in our previous publication [22]; here we concentrate on MultiSETTER only.

### Input

Structures to be aligned are supplied as the list of PDB IDs (Fig. 2b), as the file containing the list of PDB IDs separated by whitespaces or as PDB files each containing a particular structure (Fig. 2c). The last option comes handy if users need to align their own structures that were not deposited into the PDB database. Optionally, the chain ID separated by a colon from the PDB ID may be specified. Otherwise, all chains in the structure are considered for an alignment. The parameters of the SETTER algorithm for the pairwise structural alignment, described in a detail previously [21, 22], can be easily modified on the input page (Fig. 2d). However, in such case only the $\overline{S}$-distance is reported because *p*-value calculation is possible only for default algorithm parameters. When a user submits a query, the SETTER database is checked for the presence of structures the query consists of. If a structure is not present in the SETTER database, it is downloaded from the PDB database [43], parsed, its hydrogen-bonding patterns are calculated by 3DNA [42] and the whole structure with all metadata is stored in the SETTER database. From the list of structures and their metadata, the XML file that carries the configuration of a multiple structure alignment task is generated and sent to the MultiSETTER application. The application performs the calculation specified in the XML file in the background. Once finished, an output XML file with the results is created and further processed to visualize the results. A user is notified on a screen (Fig. 2e) about all steps of the process. In the case of a successful alignment, a user is provided with a unique URL that is valid for the next 10 days.

### Output

**Fig. 3:**
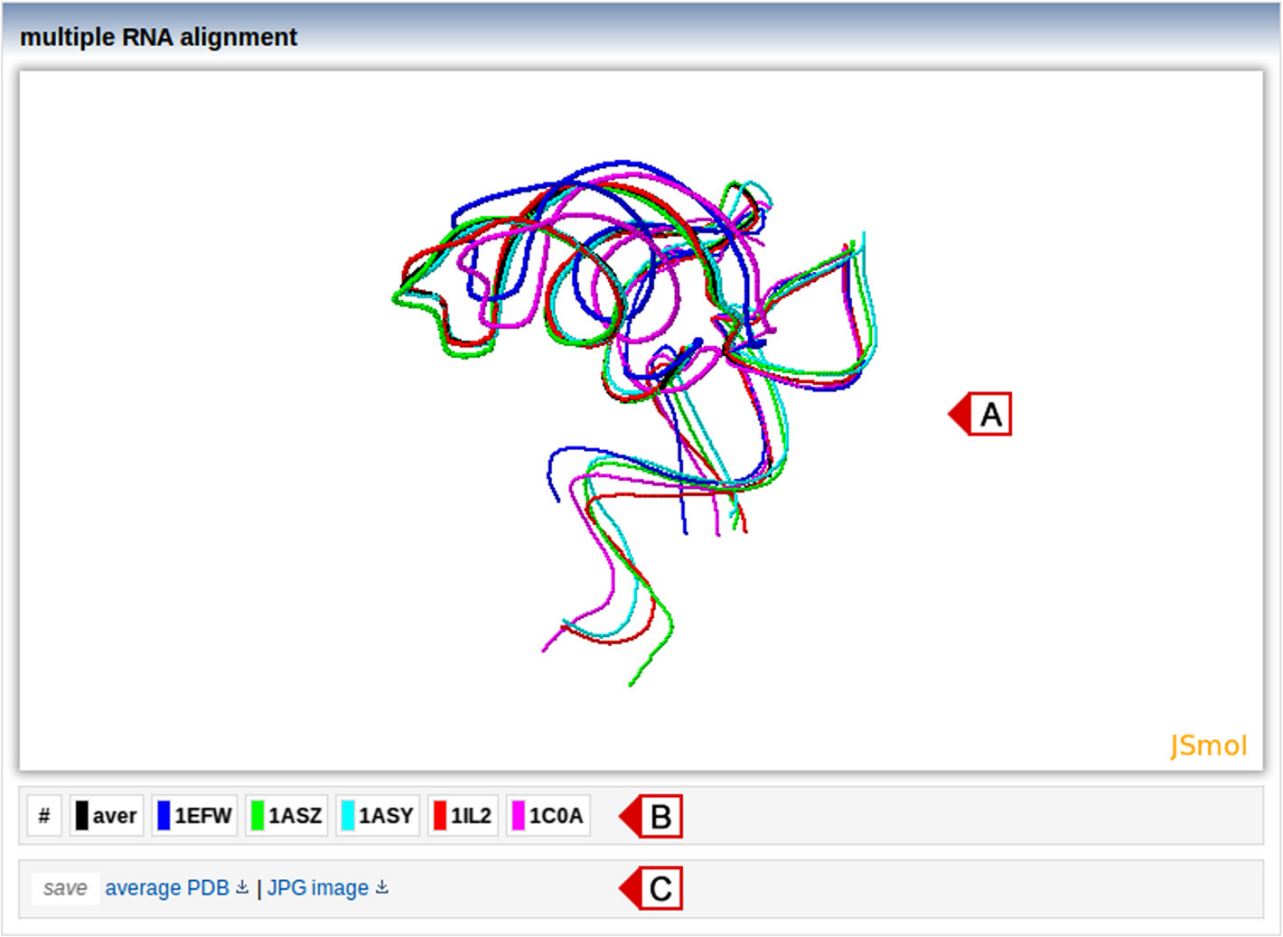
Multiple alignment view. **a** Aligned structures are visualized using the JavaScript-based molecular viewer JSmol. The average structure is depicted in black. **b** The controls of RNA structure visibility. **c** A link to save an average structure as a PDB file and a link to take the JPG snapshot of the current view

Alignment results are displayed in the multiple alignment view under the ‘results’ tab (Fig. 3a). The average structure is depicted in black and individual RNA structures are displayed in color. The visibility of each structure can be changed by clicking at corresponding structure ID below the main window (Fig. 3b). The snapshot of a current view can be saved as JPG image and, in addition, the average structure can be downloaded as a PDB file (Fig. 3c).

**Fig. 4:**
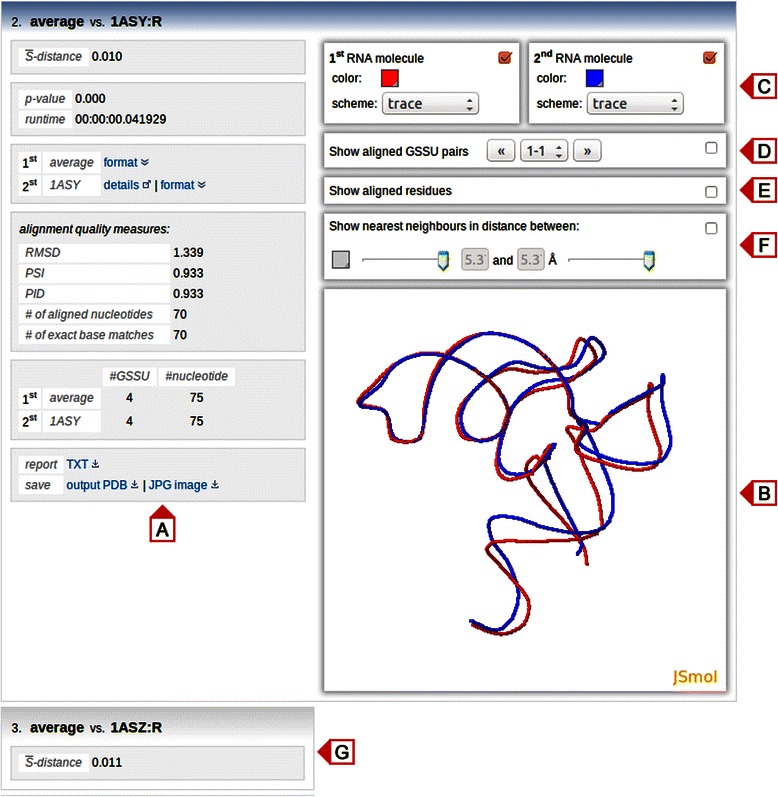
Multiple alignment view. **a** Left column contains various details about the alignment. **b** Aligned structures are visualized using JSmol. **c** The color and scheme of the visualization can be changed. **d** Aligned GSSU pairs can be displayed and either cycled by clicking left/right arrows or selected from a drop-down box. **e** The visualization of aligned residues can be turned on or off. **f** Nearest neighbor nucleotides lying within a user adjustable distance range can be highlighted. **g** Results of other pairwise alignments are displayed as vertically stacked boxes

Below the multiple alignment view, pairwise alignments between the average structure and given RNA structure are displayed as vertically stacked boxes (Fig. 4g). The boxes are ordered in a descending order by the $\overline{S}$-distances and they are, with the exception of the top box, collapsed and can be expanded by a single mouse click. Each expanded box consists of two columns. The left column (Fig. 3a) contains an $\overline{S}$-distances, a statistical significance of the alignment given as its *p*-value and a running time of the algorithm. Under the ‘format’ link, the average structure can be downloaded in the PDB format. If the aligned RNA structure is available in the PDB database, the ‘details’ hyperlink leads to its record in the PDB database, the structure can be downloaded either in PDB or in mmCIF [44] formats under the ‘format’ link and details about its primary publication are revealed by hovering over the PDB code. Several commonly used alignment quality measures are reported in the ‘alignment quality measures’ panel. These include the root-mean-square deviation between the positions of phosphate atoms, PSI (the percentage of superimposed residues within 4.0 Å with respect to the length of the shorter of the two structures), PID (the percentage of aligned nucleotides of the same type with respect to the length of the shorter of the two structures) [13, 12], number of aligned nucleotides and number of exact base matches [17]. A table below the ‘alignment quality measures’ panel displays the number of GSSU units and the number of nucleotides in each structure. Finally, superimposed structures can be downloaded in the PDB format, the visualization of the aligned structures can be saved as a JPG image and the alignment report can be downloaded as a plain text (Fig. 4a). The right column contains the JSmol visualization of two superimposed structures (Fig. 4b). Implicitly, the average structure is shown in red and the RNA structure in blue. However, display colors, as well as molecular display schemes, can be easily adjusted (Fig. 4c). In addition, the visualization of each structure can be turned on or off. To enhance the interpretation of the alignments, the following three panels show aligned GSSU pairs (Fig. 4d), aligned residues (Fig. 4e) [22] and neighbor nucleotides within an user adjustable distance range (Fig. 4f). In the case of aligned GSSU pairs panel, two aligned GSSUs are shown in red and blue and can be cycled by clicking left/right arrows or selected from a drop-down box.

**Fig. 5:**
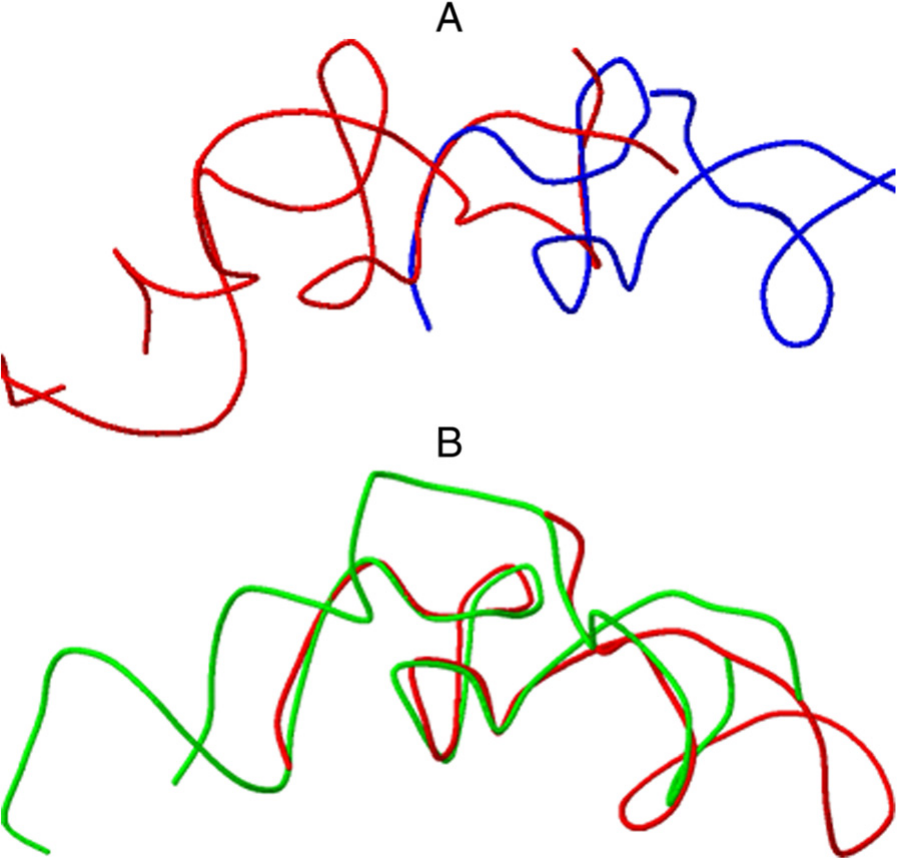
The structure alignment of 4ABR [46] and 1P6V [47] structures belonging to the RF00023 Rfam family. **a** Pairwise structure alignment of 4ABR and 1P6V produced by SETTER. **b** The alignment of 4ABR and 1P6V taken from the multiple structure alignment of the whole RF00023 family produced by MultiSETTER

The ability of a multiple structure alignment to produce better alignment than a pairwise structure alignment can be demonstrated on two structures from the Rfam [45] RF00023 family. This family contains 9 structures of a transfer-messenger RNA (tmRNA), a bacterial RNA with dual tRNA-like and mRNA-like properties. The pairwise alignment of two structures from the RF00023 family (4ABR [46] and 1P6V [47] is clearly inferior (Fig. 5a) to the alignment taken from the multiple structure alignment (Fig. 5b) of the whole RF00023 family. Thus, using the multiple structure alignment may lead to the detection of conserved regions not detected by a pairwise alignment.

## Conclusions

To the best of our knowledge, the MultiSETTER web server is the first publicly available tool for a multiple RNA structure alignment. It is built on MultiSETTER method [27] which is the extension of SETTER, our algorithm for fast and accurate RNA pairwise structure alignment [21]. Thus, the MultiSETTER server produces accurate multiple alignments in a reasonable amount of time even for largest RNA structures. Though multiple structure alignment can be performed at the secondary structure level by tools such as RNADistance from the ViennaRNA package [48], the MultiSETTER server offers the visual inspection of the alignment in 3D space which may reveal structural and functional relationships not captured by the secondary structure motifs. Further work is currently underway to apply MultiSETTER for the development of the automated system for the RNA structure classification. In addition, we will combine sequence and structure alignments in the MultiSETTER web server as we expect higher accuracy of such approach.

## Availability

**Project name:** MultiSETTER web server

**Project home page:** http://setter.projekty.ms.mff.cuni.cz/

**Operating system(s):** Platform independent

**Programming language:** Web server is developed in Python web development framework Django version 1.4. Server-side scripts are written in Python programming language version 2.7. JavaScript is required to visualize RNA structures and their alignments.

**License:** The website is free and open to all users; there is no login requirement

**Any restrictions to use by non-academics:** None
